# Barriers to and Facilitators of COVID-19 Prevention Behaviors Among North Carolina Residents

**DOI:** 10.1177/10901981221076408

**Published:** 2022-02-22

**Authors:** Lauren M. Hill, Hunter Davis, Maura Drewry, Victoria Shelus, Sophia M. Bartels, Katherine Gora Combs, Kurt M. Ribisl, Allison J. Lazard

**Affiliations:** 1 The University of North Carolina at Chapel Hill, Chapel Hill, NC, USA

**Keywords:** COVID-19, prevention, social distancing, masks, North Carolina

## Abstract

COVID-19 was the third leading cause of death in the United States in 2020. Prior to the wide dissemination of SARS-CoV-2 vaccines, individual prevention behaviors, such as wearing face masks, have been the primary non-pharmaceutical interventions to reduce infections. We surveyed 404 North Carolina residents recruited through Amazon MTurk in July 2020 to assess adherence to key prevention behaviors (6-foot distancing, mask wearing, and gathering limits) and barriers to and facilitators of adherence. Participants reported past 7-day prevention behaviors and behavioral barriers and facilitators informed by the Integrated Behavior Model and the Health Belief Model (perceived risk, perceived severity, behavioral attitudes, injunctive and descriptive norms, and personal agency). Reported adherence to each behavior in the past 7 days was generally high, with lower adherence to 6-foot distancing and mask wearing in the work context. The most commonly endorsed barriers to 6-foot distancing included physical impediments, forgetting, and unfavorable descriptive norms. For mask wearing, ability to keep a distance, discomfort/inconvenience, and forgetting were most commonly endorsed. In logistic regression models, injunctive social norms followed by perceived personal agency were the strongest independent correlates of 6-foot distancing. Behavioral attitudes and injunctive social norms were independently associated with mask wearing. For gathering size limit adherence, perceived personal agency was the strongest independent predictor followed by perceived severity of COVID-19. Messaging campaigns targeting these barriers and facilitators should be tested. Interventions improving the convenience and salience of physical distancing and mask wearing in high-density public places and places of work may also promote prevention behaviors.

COVID-19 was the third leading cause of mortality in the United States in 2020 with an estimated 375,000 deaths attributed to the disease (Ahmad et al., 2021; Centers for Disease Control and Prevention [CDC], 2020a; Reuters Staff, 2020). Like the rest of the United States, North Carolina experienced a steady increase in cases through 2020, with more than 300,000 confirmed cases and nearly 5,000 deaths over the course of the year (North Carolina Department of Health and Human Services [NC DHHS], 2020). Unfortunately, Black and Latino/a residents have faced disproportionately high case and fatality rates (Arias, 2021; NC DHHS, 2020). Due to elevated per capita case rates and test positivity rates, North Carolina has repeatedly been designated as a COVID-19 “red zone” state by the White House Coronavirus Task Force (Bonner, 2020; Kummerer & Green, 2020).

Before the arrival of an effective, approved, and broadly disseminated vaccine for COVID, behavioral prevention strategies were key non-pharmaceutical interventions (NPIs) before vaccines were given Emergency Use Authorization in December 2020 (Imai et al., 2020). Even with approved vaccines, NPIs have remained critical as much of the population remains unvaccinated as rollout continues, and the available vaccines are not yet approved for children under 16 (Food and Drug Administration [FDA], n.d.). Key NPIs recommended over the course of the pandemic have included physical distancing, masks, and limiting gathering sizes. Early evidence suggested that maintaining a distance of at least 6 feet from others was an effective measure to prevent the aerial transmission of SARS-CoV-2, although this distance has been debated (Bahl et al., 2020; Bourouiba, 2020; Chu et al., 2020). In addition to physical distancing, wearing a face mask or other covering over the nose and mouth by both symptomatic and asymptomatic members of the public can significantly reduce the risk of viral transmission (Anfinrud et al., 2020; Kai et al., 2020; Leung et al., 2020), and can effectively prevent community spread of COVID-19 (Cheng et al., 2020; Kai et al., 2020). Indeed, mask wearing and 6-foot distancing make up two key pillars of the Wear Wait Wash (three Ws) guidelines promoted by the NC DHHS and other state and local agencies (North Carolina Government, n.d.-a). Moreover, Executive Order No. 141, issued by North Carolina’s governor on May 20, 2020, and subsequent executive orders required that North Carolina residents “follow social distancing recommendations, including that everyone wear a cloth face covering, wait six (6) feet apart and avoid close contact . . .” (North Carolina Executive Order No. 141, 2020). Following evidence of large social gatherings leading to “super-spreader” events (Aschwanden, 2020; Majra et al., n.d.), limiting social gathering sizes was also a key strategy employed by North Carolina and other states to prevent the spread of COVID-19. At the time of the study, North Carolina executive orders limited indoor gatherings to a 10-person maximum, and outdoor gatherings to 25 people (North Carolina Government, n.d.-b).

Despite guidelines and mandates for these key COVID-19 prevention behaviors (6-foot distancing, mask wearing, limiting gathering size) and the devastating impact of the pandemic, media reports and escalating case numbers suggested that adherence to these recommended behaviors was mixed (Coroiu et al., 2020; Haischer et al., 2020; Katz et al., 2020). Despite substantial evidence of gaps in adherence, we have little empirical understanding of these gaps as well as of barriers to and facilitators of adherence. One international study identified key barriers to social distancing in the early phase of the pandemic, including family responsibilities and negative social norms toward distancing (Coroiu et al., 2020). Other studies have described adherence to prevention behaviors in the United States but have not assessed barriers to adherence (Park et al., 2020; Parsons Leigh et al., 2020). There is some evidence that suggests demographic differences in adherence, with rural residents and Republican-identifying individuals having a lower likelihood of mask wearing (Kramer, 2020; UNC Public Policy Graduates and Students, 2020).

To improve efforts to control the COVID-19 pandemic in North Carolina and the United States, we need to understand what modifiable barriers and facilitators will help increase COVID-19 prevention behaviors at the population level. To this end, we conducted a survey of North Carolina residents to describe adherence to COVID-19 prevention behaviors, and to assess intra- and interpersonal factors associated with adherence including self-reported barriers to these behaviors. The results of this study highlight targetable barriers to and facilitators of behavior change communication and interventions which may further our efforts to control the COVID-19 pandemic and future respiratory infectious disease epidemics.

## Methods

### Study Context

In March 2020, an interdisciplinary research team was assembled to assist the NC DHHS in developing messaging to promote COVID-19 prevention behaviors. To inform the development of these messages, we conducted a series of online surveys of North Carolina residents recruited via Amazon Mechanical Turk (MTurk). Our agile process for message development and testing has been previously described (Bartels et al., 2021). The data presented here are from the fourth survey conducted by this group in July 2020, which focused on better understanding barriers to and facilitators of key COVID-prevention measures of priority. These included adherence to 6-foot distancing recommendations, mask wearing in public, and avoiding large social gatherings.

### Data Collection

In all, 404 North Carolina residents were recruited on a convenience basis to participate in an anonymous survey via Amazon MTurk and self-administered the survey online using the Qualtrics platform. To be eligible to participate, individuals had to report being a current North Carolina resident and at least 18 years old. The target sample size of approximately 400 participants was chosen because of the feasibility of recruitment within our study budget and time frame (this was part of a rapid data collection effort in support with the NC DHHS’ development of COVID-prevention messaging [Bartels et al., 2021]).

#### Descriptive Measures

Participants reported their age, race and ethnicity, household income, education, residence setting (rural, suburban, or urban), current work status, and political affiliation. They also reported whether they had had a positive COVID-19 test or otherwise suspected that they had had a COVID-19 infection but had no confirmatory test.

#### COVID-19 Prevention Behaviors

Participants were asked to self-report three key COVID-19 prevention behaviors in the past 7 days. For *6-foot distancing*, participants reported how much of the time they kept a distance of 6 feet or more from non-household members in three contexts: at work (if applicable), in an indoor setting away from home, and in an outdoor setting away from home. For *mask wearing*, participants reported how much of the time they wore a covering over their mouth and nose in three contexts: at work (if applicable), in public settings where they were able to keep a 6-foot distance from others, and in public settings where they were unable to keep a 6-foot distance from others. Participants rated each item on a 4-point scale (*never* to *all of the time*). We categorized responses of “most” or “all of the time” as adherent for each behavior in each relevant context. We also asked participants about their attendance of *social gatherings* with non-household members, asking them to report the size of the indoor and outdoor gatherings they had attended, if any. We categorized participants as being adherent with avoiding “large” social gatherings if they attended no gatherings exceeding the limits set by state executive orders at the time the survey was administered (10 for indoor and 25 for outdoor).

#### Directly Reported Barriers to COVID-19 Prevention Behaviors

Participants who reported not adhering to 6-foot distancing and mask wearing “all of the time” in each of the relevant contexts were asked to report barriers to each behavior through a multiple-choice question (see Table 4 in “Results” for response options).

#### Hypothesized Facilitators of COVID-19 Prevention Behaviors

We also measured six cognitive and normative constructs that we hypothesized would promote COVID-19 prevention behaviors, informed by the Integrated Behavior Model and the Health Belief Model (Fishbein, 2011; Fishbein et al., 2001; Janz & Becker, 1984): perceived risk, perceived severity, behavioral attitudes, injunctive and descriptive norms, and personal agency. Participants who did not report a prior COVID-19 diagnosis rated their *perceived risk* for becoming infected in the next year (4-point response scale from *no chance* to *high chance*). They also rated the *perceived severity* of being infected with COVID-19, based on either their own experience of infection or anticipated severity among those not reporting a known or suspected infection (4-point response scale from *not bad* to *very bad*).

For each COVID-19 prevention behavior, participants rated their agreement with statements regarding their attitude toward the behavior, perceived social norms for the behavior, and their personal agency over the behavior, rated on a 5-point Likert-type scale. *Behavioral attitude* statements reflected the perceived importance of each prevention behavior (“Staying at least 6 feet apart from people you do not live with is important to prevent the spread of the coronavirus”; “Wearing a covering over your mouth and nose [face mask] when you are in public is important to prevent the spread of the coronavirus”; “Avoiding large gatherings is important to prevent the spread of the coronavirus”). *Injunctive norm* statements addressed the perceived approval for each behavior in participants’ social circles (“Most people I know believe that we all should [stay 6 feet away from others/wear face masks/avoid hosting or attending large gatherings] to prevent the spread of the coronavirus”). *Descriptive norm* statements pertained to perceived local adherence levels with each behavior (“Most people I see in public [stay 6 feet away from others/wear a face mask] most or all of the time”; “Most people I know avoid hosting or attending large gatherings”). Finally, *personal agency* statements reflected the perceived ease of performing each behavior (“It is easy to [keep a distance of 6 feet or more from others in public spaces/wear a face mask when I am in public/avoid large gatherings]”).

### Ethical Considerations

All study procedures were approved by the institutional review board of the University of North Carolina at Chapel Hill (IRB #20-0995). All participants completed electronic informed consent prior to completing the survey.

### Data Analysis

Analyses were completed in SAS v 9.4. We first generated statistics describing the sociodemographic composition of the sample, adherence to the three COVID-19 prevention behaviors of interest, and reported barriers to and hypothesized facilitators of each behavior (frequencies and percentages of categorical variables, mean, and range of continuous variables). We then assessed the hypotheses that North Carolina residents reporting higher/more positive levels of each facilitator would be more likely to adhere to each prevention behavior than those reporting lower/less positive levels of each facilitator (dichotomized to indicate those reporting the highest levels of each predictor—see Table 5). To do this, we estimated unadjusted and adjusted odds ratios (ORs) of the association between each hypothesized facilitator and behavior of interest and corresponding 95% Wald chi-square confidence intervals (CIs). Adjusted logistic regression models included all hypothesized facilitators for the relevant behavior as independent variables. All analyses were performed with complete cases.

## Results

A total of 404 North Carolina residents completed the survey from July 23 to 25, 2020. Participants had an average age of 38 years (*SD*: 12.1 range: 18–69; Table 1). The majority of participants were non-Hispanic White (67%) followed by non-Hispanic Black or African American (14%). Participants most commonly resided in suburban locations (42%) followed by urban residence (35%). Regarding employment, participants were most commonly working remotely (40%), 33% were working in-person some or all of the time, and 20% were unemployed or furloughed. Finally, 8% of participants reported having tested positive for SARS-CoV-2 while 11% suspected they may have had COVID-19 but never had a test to confirm.

**Table 1.** Descriptive Sociodemographic and SARS-CoV-2 Infection Statistics.

Variable	*n* (%) or *M* ± *SD* (range)
Age (years)	38 ± 12.1 (18–69)
Gender (*n* = 402)	
Male	194 (48.3%)
Female	207 (51.5%)
Other	1 (0.3%)
Race/ethnicity (*n* = 398)	
Non-Hispanic White	268 (67.3%)
Hispanic White	21 (5.3%)
Non-Hispanic Black/African American	55 (13.8%)
Hispanic Black/African American	29 (7.3%)
Asian or Pacific Islander	15 (3.8%)
Other (including multiple races)	10 (2.5%)
Highest educational attainment (*n* = 401)	
High school or less	34 (8.5%)
Some college or technical school	97 (24.2%)
Bachelor’s degree	201 (50.1%)
Graduate or professional degree	69 (17.2%)
Household income (past 12 months) (*n* = 401)	
Less than US$25,000	71 (17.7%)
US$25,000–US$49,999	115 (28.7%)
US$50,000–US$74,999	103 (25.7%)
US$75,000 or more	112 (27.9%)
Residence location (*n* = 401)	
Urban	140 (34.9%)
Suburban	170 (42.4%)
Rural	91 (22.7%)
Political affiliation (*n* = 401)	
Democrat	135 (33.7%)
Independent	154 (38.4%)
Republication	112 (27.9%)
Work status (*n* = 394)	
Unemployed or furloughed	80 (19.8%)
Working remotely only	161 (39.9%)
Working at place of work some or all of the time	135 (33.4%)
Retired	18 (4.5%)
SARS-CoV-2 infection in past year (*n* = 404)	
Yes	33 (8.2%)
Maybe	44 (10.9%)
No	327 (80.9%)

### Reported COVID-19 Prevention Behaviors

The majority of participants reported keeping a 6-foot distance from non-household members most or all of the time in the past 7 days (Table 2). In all, 80% and 79% of respondents reported keeping a 6-foot distance from others outside of the home in indoor and outdoor contexts, respectively. Yet, just under 70% of respondents working in-person reported keeping a distance from others while at work. A similar pattern was seen in reported mask wearing. A large majority—82% and 85%—of respondents reported wearing a mask when they were able and unable to keep a 6-foot distance from others, respectively. A total of 78% of applicable respondents reported wearing a mask while at work.

**Table 2.** COVID-19 Prevention Behaviors in the Past 7 Days (*n* = 404).^a^

Past week risk-reduction practices	*n* (%) reporting		
6-feet distancing	At work (if applicable) *n =* 135	Indoors, away from home	Outdoors, away from home
Never	5 (3.7%)	21 (5.2%)	19 (4.7%)
Some of the time	36 (26.7%)	58 (14.4%)	67 (16.6%)
Most of the time	74 (54.8%)	167 (41.3%)	155 (38.4%)
All of the time	20 (14.8%)	158 (39.1%)	163 (40.3%)
Adherent (most or all of the time)	94 (69.6%)	325 (80.4%)	318 (78.7%)
Mask wearing	At work (if applicable) *n =* 134	Away from home, able to keep 6-feet distance *n =* 403	Away from home, unable to keep 6-feet distance *n =* 403
Never	16 (11.9%)	15 (3.7%)	16 (4.0%)
Some of the time	13 (9.7%)	59 (14.6%)	44 (10.9%)
Most of the time	45 (33.6%)	138 (34.2%)	94 (23.3%)
All of the time	60 (44.8%)	191 (47.4%)	249 (61.8%)
Adherent (most or all of the time)	105 (78.4%)	329 (81.6%)	343 (85.1%)

a *n* = 404 unless otherwise noted.

About one half of respondents had attended a social gathering of any size in the past 7 days (52% attended indoor gatherings, 46% attended outdoor gatherings; Table 3). A total of 22% reported attending indoor gatherings exceeding 10 people in size. A total of 12% reported attending outdoor gatherings exceeding 25 people in size.

**Table 3.** Social Gathering Size in the Past 7 days (*n* = 403).

Social gathering attendance	*n* (%) reporting
Size of largest indoor gathering	
Did not attend indoor gathering	193 (47.9%)
2–10 people	122 (30.3%)
11–25 people	42 (10.4%)
More than 25 people	46 (11.4%)
Size of largest outdoor gathering	
Did not attend indoor gathering	217 (53.9%)
2–10 people	94 (23.3%)
11–25 people	45 (11.2%)
More than 25 people	47 (11.7%)
Adherence to gathering size guidelines	
Indoor gatherings only ≤10	315 (78.2%)
Outdoor gatherings only ≤25	356 (88.3%)
Adherent with both	308 (76.4%)

### Reported Barriers to COVID-19 Prevention Behaviors

The 327 individuals who reported that they do not always adhere to 6-foot distancing guidelines were asked directly about barriers to physical distancing (Table 4). By far the most common response, 76% of participants endorsed the statement “some places or activities make it [6-foot distancing] difficult.” Second most common was “I forget to keep a distance,” which was endorsed by 31% of participants. Reflecting social norms, 24% reported “most people are not doing this [keeping a distance]” and 17% endorsed “I’m concerned what others will think of me if I keep a distance.” A total of 16% of participants doubted the efficacy of physical distancing (“I doubt keeping a distance of 6 feet from others will protect me or others from the coronavirus”) and 15% reported physical-distancing fatigue (“I’m tired of staying 6 feet away from people”). All other responses were endorsed by fewer than 15% of participants.

**Table 4.** Directly Reported Barriers to COVID-19 Prevention Behaviors.

Reported barriers by practice	*n* (%)
6-feet distancing, *n* = 327 What are the reasons that you don’t always keep a distance of 6 feet or more from others?	
Some spaces or activities make it difficult	248 (75.8%)
I forget to keep a distance	101 (30.9%)
Most people are not doing this	77 (23.6%)
I’m concerned what others will think of me if I keep a distance	57 (17.4%)
I doubt keeping a distance of 6 feet from others will protect me or others from the coronavirus	51 (15.6%)
I’m tired of staying 6 feet away from people	50 (15.3%)
The government can’t force me to do this	33 (10.1%)
Other	37 (11.3%)
Mask wearing, *n* = 267 What are the reasons that you sometimes do not wear a covering over your mouth and nose (face mask) when you leave your home?	
I don’t come close enough to other people to need a mask	127 (47.6%)
It is uncomfortable or inconvenient to wear a mask	82 (30.7%)
I forget to bring/wear a mask	77 (28.8%)
I doubt wearing a mask will protect me or others from the coronavirus	53 (19.9%)
I’m not sick/I don’t have COVID-19 symptoms	51 (19.1%)
Most people are not wearing masks	46 (17.2%)
I’m concerned what others will think of me or do to me if I wear a mask	37 (13.9%)
The government can’t force me to wear a mask	35 (13.1%)
I don’t understand when I should wear a mask	26 (9.7%)
I don’t own a mask	18 (6.7%)
Other	31 (11.6%)

The 267 individuals who reported not always wearing a mask when away from home were also asked directly about barriers to mask wearing. The most common response was “I don’t come close enough to other people to need a mask” (48% of participants). Convenience issues were also common; 31% reported “It is uncomfortable or inconvenient to wear a mask” and 29% reported “I forget to bring/wear a mask.” Efficacy doubts were also reported by 20% of participants (“I doubt wearing a mask will protect me or others from the coronavirus”) and 19% reported not wearing a mask because “I’m not sick/I don’t have COVID-19 symptoms.” Norm-related barriers were also reported by some participants: 17% said they did not always wear a mask because “most people are not wearing masks” and 14% were “concerned what others will think of me or do to me if I wear a mask.” All other responses were endorsed by fewer than 14% of participants.

### Hypothesized Facilitators of COVID-19 Prevention Behaviors

Among the 372 participants who had not had a positive SARS-CoV-2 test, 47% believed they had a small chance of being infected in the next year while 39% believed they had a medium chance (Table 5). When asked about the perceived severity of a COVID-19 infection, participants most commonly responded that being infected would be (or had been) “moderately bad” (38%).

**Table 5.** Hypothesized Facilitators and Associations With COVID-19 Prevention Behaviors Adherence (*n* = 404).^a^

Reported behavioral predictor	6-feet distancing adherence			Mask wearing adherence			Avoiding large gatherings adherence		
	*n* (%)	OR (95% CI)	aOR (95% CI)	*n* (%)	OR (95% CI)	aOR (95% CI)	*n* (%)	OR (95% CI)	aOR (95% CI)
Perceived risk (1-year perceived COVID infection risk)^b^	*n* = 371			—			—		
No chance	28 (7.5%)								
Small chance	175 (47.2%)								
Medium chance	146 (39.1%)								
High chance	23 (6.2%)								
		1.81	1.7		1.75	1.48		1.69	1.47
Medium or high chance	168 (45.3%)	[1.17, 2.78]**	[1.07, 2.71]		[1.12, 2.75]*	[0.91, 2.40]		(0.99, 2.89)	(0.84, 2.57)
Perceived severity (of COVID infection)^b^				—			—		
Not bad	40 (9.9%)								
Slightly bad	114 (28.2%)								
Moderately bad	152 (37.6%)								
Very bad	98 (24.3%)								
		1.66	1.37		1.86	1.63		1.74	1.77
Moderate or very bad	250 (61.9%)	[1.10, 2.51]*	[0.85, 2.21]		[1.21, 2.85]**	[1.00, 2.66]		(1.09, 2.77)*	(1.02, 3.08)*
Behavioral attitude (perceived importance)				*n* = 403			*n* = 403		
Strongly disagree	3 (0.7%)			5 (1.2%)			5 (1.2%)		
Disagree	8 (2.0%)			14 (3.5%)			7 (1.7%)		
Neither agree nor disagree	38 (9.4%)			35 (8.7%)			32 (7.9%)		
Agree	138 (34.2%)			105 (26.1%)			126 (31.3%)		
Strongly Agree	217 (53.7%)			244 (60.6%)			233 (57.8%)		
		3.37	1.96		4.33	2.18		2.83	1.61
Agree or strongly agree	355 (87.9%)	[1.81, 6.27]**	[0.90, 4.24]	349 (86.6%)	[2.38, 7.85]***	[1.07, 4.45]*	359 (89.1%)	[1.48, 5.41]**	[0.71, 3.63]
Injunctive social norms				*n* = 403			*n* = 403		
Strongly disagree	6 (1.5%)			9 (2.2%)			6 (1.5%)		
Disagree	30 (7.4%)			41 (10.2%)			23 (5.7%)		
Neither agree nor disagree	59 (14.6%)			46 (11.4%)			59 (14.6%)		
Agree	174 (43.1%)			155 (38.5%)			177 (43.9%)		
Strongly agree	135 (33.42%)			152 (37.7%)			138 (34.2%)		
		3.23	2.27		2.94	2.05		1.5	0.82
Agree or strongly agree	309 (76.5%)	[2.01, 5.20]***	[1.31, 3.92]**	307 (76.2%)	[1.83, 4.72]***	[1.15, 3.65]*	315 (78.2%)	[0.88, 2.54]	[0.38, 1.78]
Descriptive social norms				*n* = 403			*n =* 403		
Strongly disagree	25 (6.2%)			10 (2.5%)			5 (1.2%)		
Disagree	93 (23.0%)			57 (14.1%)			31 (7.7%)		
Neither agree nor disagree	65 (16.1%)			76 (18.9%)			56 (13.9%)		
Agree	151 (37.4%)			183 (45.4%)			184 (45.7%)		
Strongly Agree	70 (17.3%)			77 (19.1%)			127 (31.5%)		
		1.22	0.77		1.66	1.25		1.59	1.61
Agree or strongly agree	221 (54.7%)	[0.81, 1.82]	[0.47, 1.26]	260 (64.5%)	[1.08, 2.57]*	[0.76, 2.08]	311 (77.2%)	(0.95, 2.68)	(0.76, 3.41)
Perceived personal agency				*n =* 403			*n =* 403		
Strongly disagree	15 (3.7%)			2 (0.5%)			3 (0.74%)		
Disagree	72 (17.8%)			19 (4.7%)			20 (5.0%)		
Neither agree nor disagree	67 (16.6%)			35 (8.7%)			34 (8.4%)		
Agree	161 (39.9%)			154 (38.2%)			139 (34.5%)		
Strongly agree	89 (22.0%)			193 (47.9%)			207 (51.4%)		
		1.99	1.78		2.33	1.35		1.95	2.54
Agree or strongly agree	250 (61.9%)	[1.31, 3.01]**	[1.10, 2.91]*	347 (86.1%)	[1.31, 4.14]**	[0.68, 2.67]	346 (85.9%)	[1.07, 3.56]*	[1.27, 5.08]**

*Note*. ORs represent association with adherence to each prevention behavior as defined in Table 2. OR = odds ratio; CI = confidence interval; aOR = adjusted odds ratio.

a *n*=404 unless otherwise noted. ^b^This predictor is not behaviorally specific, thus is described in first column only.

* *p* < .05. **p < .01. ***p < .0001.

Participants reported highly positive attitudes toward the importance of 6-foot distancing (88% agree or strongly agree), and moderately positive injunctive social norms (77% agree or strongly agree) and perceived personal agency (62% agree or strongly agree) for distancing. Lowest rated was descriptive social norms, with only 55% of participants agreeing or strongly agreeing that most people they see maintain a 6-foot distance from others in public.

Regarding mask wearing, participants reported highly positive attitudes toward mask wearing (87% agree or strongly agree) and high perceived personal agency for mask wearing (86% agree or strongly agree). Respondents reported moderately positive injunctive social norms toward mask wearing (76% agree or strongly agree), but less positive descriptive social norms for mask wearing (65% agree or strongly agree).

For social gatherings, participants reported highly positive attitudes toward avoiding large social gatherings (89% agree or strongly agree) and high perceived personal agency for avoiding large gatherings (86% agree or strongly agree). Respondents also reported moderately positive injunctive social norms (78% agree or strongly agree) and descriptive social norms (77% agree or strongly agree) for avoiding large social gatherings.

### Association of Hypothesized Facilitators With Prevention Behavior Adherence

In bivariate analyses, all hypothesized predictors were associated with reported adherence to 6-foot distancing except descriptive social norms (Table 5). Most closely associated with odds of 6-foot distancing adherence were behavioral attitudes (OR = 3.37; 95% CI = [1.81, 6.27]) and injunctive social norms (OR = 3.23; 95% CI = [2.01, 5.20]). At a lower magnitude, perceived risk (OR = 1.81; 95% CI = [1.17, 2.78]), perceived severity (OR = 1.66; 95% CI = [1.10, 2.51]), and greater personal agency (OR = 1.99; 95% CI = [1.31, 3.01]) were also associated with higher odds of adherence to distancing. In adjusted analyses, only injunctive social norms (aOR = 2.27; 95% CI = [1.31, 3.92]) and personal agency (aOR = 1.78; 95% CI = [1.10, 2.91]) remained associated with 6-foot distancing adherence.

All hypothesized predictors were associated with adherence to mask wearing most or all of the time in bivariate analyses (Table 5). A higher level of perceived COVID-19 risk (OR = 1.75; 95% CI = [1.12, 2.75]) and higher perceived severity of COVID-19 infection (OR = 1.86; 95% CI = [1.21, 2.85]) were moderately associated with higher odds of mask wearing. Most closely associated with mask wearing was behavioral attitudes (OR = 4.33; 95% CI = [2.38, 7.85]) and injunctive social norms (OR = 2.94; 95% CI = [1.83, 4.72]). Descriptive social norms (OR = 1.66; 95% CI = [1.08, 2.57]) were moderately associated with odds of mask wearing, as was personal agency for mask wearing (OR = 2.33; 95% CI = [1.31, 4.14]). In adjusted analyses, only behavioral attitudes (aOR = 2.18; 95% CI = [1.07, 4.45]) and injunctive social norms (aOR = 2.05; 95% CI = [1.15, 3.65]) remained associated with mask wearing.

In contrast, only three of the hypothesized facilitators were associated with avoidance of large social gatherings in bivariate analyses. Most strongly associated with odds of adherence was behavioral attitudes (OR = 2.83; 95% CI = [1.48, 5.41]). Greater perceived severity of COVID-19 infection (OR = 1.74; 95% CI = [1.09, 2.77]) and perceived personal agency (OR = 1.95; 95% CI = [1.07, 3.56]) were moderately associated with avoiding large social gatherings. In adjusted analyses, only perceived severity (aOR = 1.77; 95% CI = [1.02, 3.08]) and perceived personal agency (aOR = 2.54; 95% CI = [1.27, 5.08]) remained associated with avoiding large social gatherings.

## Discussion

A majority of participants reported adhering to each COVID-19 prevention behavior, while 18% to 30% of respondents did not regularly adhere. While reported 6-foot physical distancing was lowest in the work context (yet still high with 70% adherence), public health and workplace safety agencies recommend mask wearing in work contexts where physical distancing is not possible (CDC, 2020b; Occupational Safety and Health Administration [OSHA], n.d.), providing a reduction in transmission risk (Chu et al., 2020). As some work environments make physical distancing difficult or impossible, innovative solutions such a staggered work schedules and reorganization of work areas are needed to enable workers and clients to maintain sufficient distance (Michaels & Wagner, 2020). This, along with facilitation of mask wearing in the workplace, may help to reduce the disproportionate burden of infections and adverse outcomes among essential workers (The Lancet Editors, 2020), who are unable to work from home and control their physical work environment. The most common barriers to physical distancing endorsed by participants included unfavorable social norms, efficacy doubts, and distancing fatigue while injunctive social norms were the strongest independent predictor of physical distancing adherence in our hypothesis tests. Other studies have found similar connections between social norms for social distancing and distancing behaviors or intentions (Frounfelker et al., 2021; Norman et al., 2020), with one study suggesting that norms for social distancing may be influential as a signal to individuals about the seriousness of COVID-19 as a public health threat (Norton et al., 2021). To best leverage injunctive norms to promote physical distancing adherence, messaging and conversations promoting physical distancing may be most influential if they are received from a person an individual perceives as sharing a common identity or being a member of the same in-group as them (Abrams et al., 1990; Centola, 2011; Van Bavel et al., 2020); thus, messages from peers and leaders of groups that individuals identify with (e.g., political and religious leaders) may be most influential.

Reported mask-wearing adherence was high overall but was also lowest in the work context, and highest in situations where participants were unable to maintain physical distancing. While these trends are aligned with recommendations, 15% of participants did not report wearing a mask most or all of the time where physical distancing was not possible. The most common self-reported barriers to mask wearing endorsed by participants included convenience or comfort issues, unfavorable social norms, efficacy doubts, and a belief that masks are only needed for people who feel sick or have COVID-19 symptoms. The latter two barriers may be related to initial miscommunication at the federal and local level regarding the importance of mask wearing among asymptomatic individuals (Asmelash, 2020; Jankowicz, 2020; Jingnan et al., 2020), although many agencies have since amended their mask recommendations. Among the hypothesized facilitators of mask wearing, positive behavioral attitudes and injunctive social norms were most strongly independently correlated with adherence. Injunctive social norms (i.e., perceived approval for masks in participants’ social circles) could be leveraged to promote mask wearing through network-based and peer-influence campaigns (e.g., social media campaigns). For example, popular opinion leader interventions have been effective tools to promote HIV-prevention and other behaviors where peer or network member opinions have a significant influence on individual attitudes and behaviors (Jones et al., 2008; Sikkema et al., 2000).

While most participants were adherent with state social gathering size limits at the time of data collection, a sizable minority—one quarter—reported nonadherence. Moreover, simple adherence to gathering size mandates may not be sufficient at a population level to minimize transmission (Sohn, 2020), and more than half of the participants reported attending an indoor gathering in the past week. In our models, perceived personal agency and perceived severity of COVID-19 were independently associated with avoiding large gatherings. Other studies have had similar findings regarding the connection between perceived severity and adherence to COVID-19 prevention behaviors and vaccine intentions (Luo et al., 2021; Ren et al., 2021; Shmueli, 2021; Yu et al., 2021). While severity of COVID-19 varies by age and comorbidity (Gallo Marin et al., 2021; Sanyaolu et al., 2020), increased exposure to COVID-19 information through traditional and social media may promote greater perceived COVID-19 severity (Li et al., 2020; Ren et al., 2021). Improved education and communication about the importance of avoiding larger social gatherings, especially indoors, may also be needed. For example, the United States has widely disseminated the three Ws (Wear, Wait, Wash), but there has been little adoption of the 3 Cs campaign (Closed spaces, Crowded places, Close contact), which was widely adopted in Japan and other Asian countries who have had greater success at containing the virus than the United States (Government of Japan, 2020; World Health Organization, n.d.).

### Limitations

The results of this study should be interpreted with key limitations in mind. First, participants were recruited using convenience sampling through MTurk and may not be representative of the population of North Carolina. Importantly, certain demographic categories including Black residents of North Carolina are noted to be underrepresented in the sample. Second, reported adherence to and attitudes toward COVID-19 prevention behaviors are likely susceptible to social desirability bias. However, this potential bias is mitigated by the fact that participants completed the survey anonymously and prompts were included to acknowledge the difficulty of perfect adherence (e.g., “We understand that it is not always possible or may be difficult to practice all of the recommendations related to coronavirus/COVID-19 and simply ask for your most accurate representation”). Third, reported adherence to each behavior is also likely susceptible to recall bias. To minimize this issue, we restricted the recall period to the past 7 days. Finally, given the cross-sectional nature of the data, causal inferences for the associations observed should be drawn with caution. Adherence to prevention behaviors reported cross-sectionally may also not reflect the level of adherence at the time of publication. Understanding of the primary barriers and facilitators associated with preventive behaviors nevertheless provides actionable and durable evidence to inform communication, programs, and structural interventions to reduce infections in this pandemic and future pandemics involving other coronaviruses and similar respiratory infectious diseases.

## Conclusion

Adherence to COVID-19 prevention behaviors was generally high among North Carolina residents surveyed in July 2020. Yet approximately one fifth to one quarter of participants were nonadherent with each protective behavior, with high levels of reported large indoor gatherings presenting a particular area of concern. Barriers reported by participants, as well as results of our models examining hypothesized facilitators of these prevention behaviors, suggest that future messaging campaigns should target efficacy beliefs for prevention behaviors, social norms, and perceived severity of COVID-19 infection. Interventions improving the convenience and salience of physical distancing and mask wearing in high-density public places (e.g., stores, restaurants, bars) may also combat key barriers to these behaviors. Future assessments are needed to better understand evolving COVID-19 prevention behaviors, attitudes, and norms.
